# Letter from a Correspondent

**Published:** 1871-06

**Authors:** 


May 28, 1871.

Drs. Powell & Goldsmith:—*Gentlemen:* I have received a printed circular from the Macon Medical Association containing a preamble and resolutions, condemning the course pursued by the majority at the last Georgia Medical Association, held at Americus, Ga., April 12, 1871, and also a communication from the Georgia Medical Society in Savannah, asking the profession to unite with them in the adoption of suitable resolutions condemnatory of the course pursued by the Georgia Medical Association at its late meeting. While I am prepared now to unite with these gentlemen in the manner suggested, having frequently conversed with some of the Trustees and a number of your citizens and physicians, upon the causes and character of the controversy, you would be surprised to learn how many of our very best medical men in the State, who know nothing of the real merits of the difficulty, and as they are now called upon to act upon a matter which involves the professional character of every regular physician in the State, I would suggest that a full history of this controversy be written and published by some of your gentlemen who are familiar with all the facts, and who can have access to the records.

Much has been whispered by certain parties calculated to prejudice those who know nothing of the merits of the controversy, against all who have opposed the course pursued by the Faculty of the Atlanta Medical College and its present Board of Trustees. The truth must now be known. Too much is involved to conceal it any longer. The intelligent and respectable portion of the profession cannot afford to allow this personal feeling to control them in matters of such great importance, involving the honor of the profession. Justice to their private character require them to learn the truth and sustain it. Unless this is done the difficulty can never end—the breach will widen and widen, and we will all be involved in error, some in one kind and some in another, and none will be left to correct it. I, for one, am unwilling for this difficulty to terminate in that manner. I am for peace, but I am unwilling to sacrifice a single principle, or the character of the humblest brother to secure its promise, for it could be but a fraud under such circumstances. If our brethren in Augusta, Savannah and Macon, are right, they must be sustained, and if wrong, they must be condemned. If error is to prevail over truth, how long would you be able to find men to vindicate the truth. Who would be willing to join a medical society or accept a chair in a medical college, if he had no security of being protected in right, and when so declared, who could respect an institution controlled by a Board of Trustees who would allow anything but truth and justice to control their action. * * * *

I am aware that some of your gentlemen have had some personal feelings and personal difficulties, but that cannot affect either way the question that has been presented to the Georgia Medical Association. If there is a personal controversy going on between the Faculty of the Atlanta Medical College, the Atlanta Academy of Medicine, the Fulton County Medical Society or members of the same, or members of the Georgia Medical Association, I agree with Dr. Logan, it should not be introduced into the Georgia Medical Association. The amendment to the charter of the Atlanta Medical College may have been conceived in personal feeling. It may have been passed through the Legislature without the knowledge and consent of the Trustees, for the purpose of carrying into effect some personal feelings. The opposition to it on the part of some of the Faculty may have been from personal feeling. The old Board of Trustees may have rejected it on account of some personal feeling. The Fulton County Medical Society may have appointed a Committee to report these facts to the Georgia Medical Association on account of some personal feeling. These are questions that should not be brought before the Association. The question for that body to consider is wasthe amendment proper. Did those who opposed it do wrong? Did the old Board of Trustees do right in rejecting it? Could they do otherwise, and do their duty to the State and the profession. Some of the members of the Georgia Medical Association, at its session held in Augusta in 1868, may have voted to repudiate the Atlanta Medical College and its students from personal feeling. The celebrated memorial written and presented to the Legislature of Georgia in which they say "that the action of this meeting held in Augusta, which repudiated the Atlanta Medical College as a regular institution, had an utter absence of all the elements of truth." "That that session of the Associetion was not the regular meeting, but one of physicians assuming to represent the medical prefession of the State, and that it was made up almost entirely of an Atlanta clique, and of members of rival schools," etc. etc. may have been written from personal feelings. And some of the delegates who were at the Association held in Savannah, may have voted for the preamble and resolutions declaring that every insinuation made against the session of the Georgia Medical Association in the celebrated memorial by the Faculty of the Atlanta Medical College to be false, may have had strong personal feeling. * * * *

Some of the gentlemen who were present in Macon in 1870, and voted for the resolution expelling the Faculty of the Atlanta Medical College, may have had some personal feeling. Dr. Logan and his party at the late meeting held at Americus, April 12, 1871, may have offered and voted for his expunging resolution from personal feeling. * * * * * *

It is immaterial with the profession whether there was or was not any personal feeling in these various actions of the Georgia Medical Association. The questions for the profession are: Was the action in Augusta just? Are the charges made in the memorial by the Faculty of the Atlanta Medical College true or false? Was the Association, in 1869, justifiable in pronouncing "every part and insinuation contained in the charge against the session held in Augusta in 1868 false—knowingly false." Under all the circumstances, could the Association held in Macon in 1870, have done less than expel the offending party? The Macon Medical Association, in their resolutions, passed recently in relation to the late action of the Georgia Medical Association, held at Americus, Ga., say they could not have done less. Knowing all the facts as I do, I cannot see how less could have been done without stultifying the Association and disgracing the profession—hence I hope every medical man in the State will be furnished with a full history of the whole controversy. A great principle is involved, which must be maintained, or the honor and dignity of the profession sacrificed.

